# Swimming droplets driven by a surface wave

**DOI:** 10.1038/srep08546

**Published:** 2015-02-24

**Authors:** Hiroyuki Ebata, Masaki Sano

**Affiliations:** 1 Department of Physics, Graduate School of Science, Chiba University, Yayoi-cho, Chiba, 263-8522, JAPAN; 2 Department of Physics, Graduate School of Science, The University of Tokyo, Hongo, Tokyo 113-0033, JAPAN

## Abstract

Self-propelling motion is ubiquitous for soft active objects such as crawling cells, active filaments, and liquid droplets moving on surfaces. Deformation and energy dissipation are required for self-propulsion of both living and non-living matter. From the perspective of physics, searching for universal laws of self-propelled motions in a dissipative environment is worthwhile, regardless of the objects' details. In this article, we propose a simple experimental system that demonstrates spontaneous migration of a droplet under uniform mechanical agitation. As we vary control parameters, spontaneous symmetry breaking occurs sequentially, and cascades of bifurcations of the motion arise. Equations describing deformable particles and hydrodynamic simulations successfully describe all of the observed motions. This system should enable us to improve our understanding of spontaneous motions of self-propelled objects.

From single cells to individual animals, living organisms spontaneously migrate by self-propelling motions such as swimming, crawling or walking. Since self-motile objects move without external force, symmetry-breaking is indispensable for self-propelled motion^1^,^2^,^3^,^4^,^5^. During revival of biological cells after drag treatment, bifurcation from a resting to a migrating state is accompanied by a change in shape^2^. To generate polarity and motility of crawling cells, actin must localize at pseudopods and tails^2^,^6^,^7^. Migration and deformation of cells are strongly connected through the actin network. By considering self-motile objects as nonlinear dynamical systems, searching for a general relationship between deformation and migration becomes worthwhile. However, in existing experimental systems, limited access to control parameters has hampered our understanding of the rich dynamics of self-propulsion.

Regardless whether a system is living or non-living, the problems concerning how deformable materials swim in a viscous fluid have attracted considerable attention for a long time^8^,^9^,^10^,^11^,^12^,^13^,^14^,^15^. A necessary condition for micro-swimmers in viscous fluids is non-reciprocal dynamics, which implies asymmetry between the forward and backward processes of deformation^8^. When the size of a swimmer exceeds *O*(mm), Stokes' approximation is weakly violated. To explore universal laws of self-propelled objects on this scale, we propose a non-living system comprising a swimming droplet in a viscous fluid. We demonstrate that a water droplet can swim on an oil bath using a surface wave through vertical vibration. As we vary the vibration frequency and viscosity of the oil, spontaneous symmetry breaking occurs sequentially, and the droplet motion exhibits cascades of bifurcations. Equations for deformable particles derived from a symmetry argument can be used to describe all of the observed motions. The droplet motion can be easily reproduced by controlling the frequency, acceleration, and viscosity. Thus, swimming droplets driven by surface waves provide a prototypical system for representing soft active particles.

## Results

### Motion of droplet on viscous fluid

**Figure 1:**
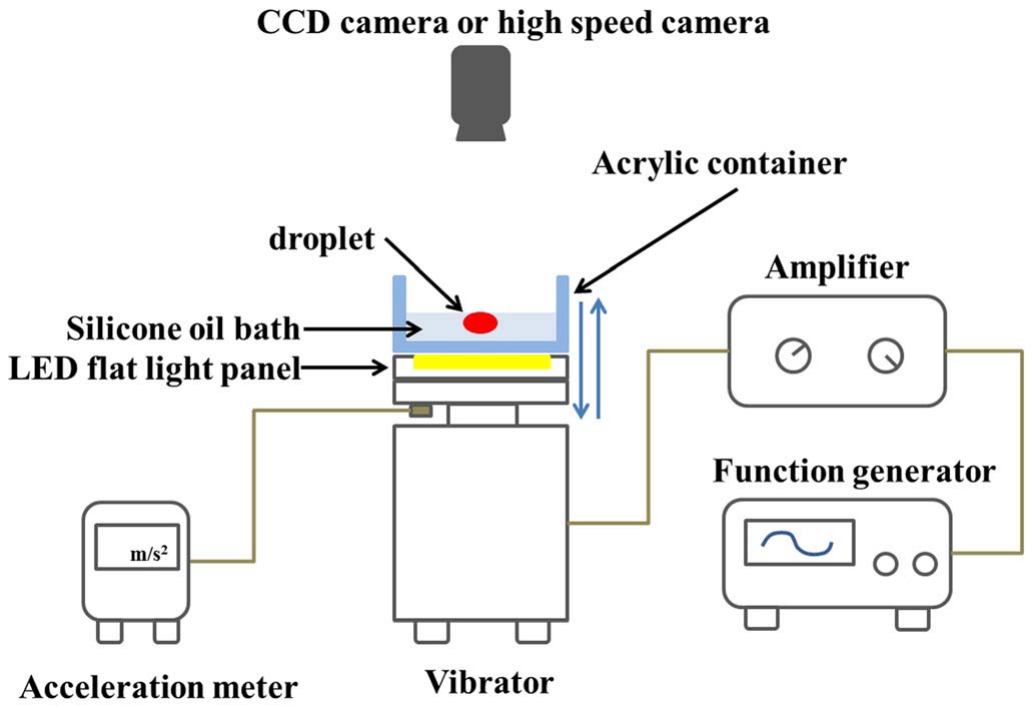
Schematic illustration of experimental setup (see Methods).

**Figure 2:**
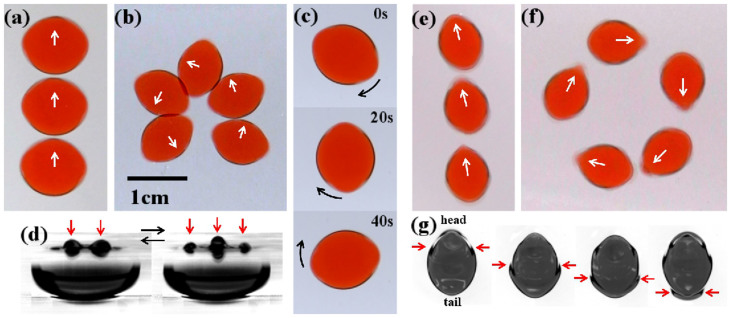
Top view of typical motion induced by standing wave. (a–c, e, f) Arrows indicate the direction of motion. Time-lapse images of (a) straight motion, (b) rotational motion, and (e, f) squirming motion. Time series of (c) spinning motion. (d) Lateral view of a droplet with spinning motion. The horizontal axis is parallel to the long axis of the droplet. Arrows indicate the antinodes, and the number of antinodes *N_a_* = 5. (g) Top-view image of squirming motion captured by a high-speed camera. The red arrow indicates the peak of the traveling wave at the triple line. The droplet migrates upward. (a) *ν* = 170 mm^2^/s, *f* = 88 Hz, *V_d_* = 0.6 ml, Γ = 75 m/s^2^. (b) *ν* = 400 mm^2^/s, *f* = 100 Hz, *V_d_* = 0.3 ml, Γ = 112 m/s^2^. (c) *ν* = 400 mm^2^/s, *f* = 80 Hz, *V_d_* = 0.6 ml, Γ = 63 m/s^2^. (d) *ν* = 400 mm^2^/s, *f* = 108 Hz, *V_d_* = 0.6 ml, Γ = 84 m/s^2^. (e, f) *ν* = 37 mm^2^/s, *f* = 120 Hz, *V_d_* = 0.3 ml, (e) Γ = 115 m/s^2^, (f) Γ = 123 m/s^2^. (g) *ν* = 37 mm^2^/s, *f* = 100 Hz, *V_d_* = 0.66 ml, Γ = 79 m/s^2^.

A water droplet is deposited and floats on a layer of highly viscous silicone oil (1 cm deep) in an acrylic container (9 cm inner diameter). Figure 1 shows a schematic illustration of the experimental setup. Because of the small difference in density between water and silicone oil (approximately 0.06 g/cm^3^) and the large surface tension, a water droplet forms a distorted hemisphere that almost sinks into the silicone oil (Fig. 2(d)). A vertical sinusoidal vibration with frequency *f* is applied to form a spatially uniform and time-symmetric agitation (the vertical position of container is *z*(*t*) = *A*sin (2*π* *f t*)). When the acceleration of vibration *Γ = A*(2*π* *f*)^2^ exceeds a critical value, a resonance occurs at the droplet-air interface, and a standing wave (Faraday wave) appears on the stationary circular droplet^16^,^17^. The Faraday wave always forms a parallel standing wave (antinode is indicated by the red arrow in Fig. 2(d)), and the droplet is forced to elongate^16^,^17^. Since silicone oil has much higher viscosity than the droplet, the Faraday wave only appears at the air-droplet interface for all of the parameters we examined. The elongated droplet undergoes various motions depending on the parameters used:

### Straight motion

The droplet undergoes translational motion. The trajectory of the centroid is a straight line (Fig. 2(a)).

### Rotational motion

The droplet undergoes translational motion. The trajectory of the centroid is a circle (Fig. 2(b)). Both the long axis and the direction of the velocity constantly rotate at the same speed, and the droplet always migrates along the short axis^17^.

### Spinning motion

The centroid of the droplet does not move, but the long axis of the droplet rotates at a constant speed (Fig. 2(c)).

### Squirming motion

When the silicone-oil bath is less viscous, the droplet migrates along the long axis depending on *f* and *Γ* (Figs. 2(e) and (f)). In this case, a traveling wave is generated at the water-oil-air triple line. The traveling wave periodically propagates from the “head” to the “tail” of the droplet at nearly the same frequency as the Faraday wave (the traveling wave is indicated by the red arrow in Fig. 2(g); see also the Supplemental Information (SI)). The droplet migrates in the direction opposite the traveling wave.

The Reynolds number (*Re*) of straight, rotational, and spinning motion is approximately 0.1. The *Re* of squirming motion is approximately 10.

### Swimming mechanism of droplets due to surface wave

**Figure 3:**
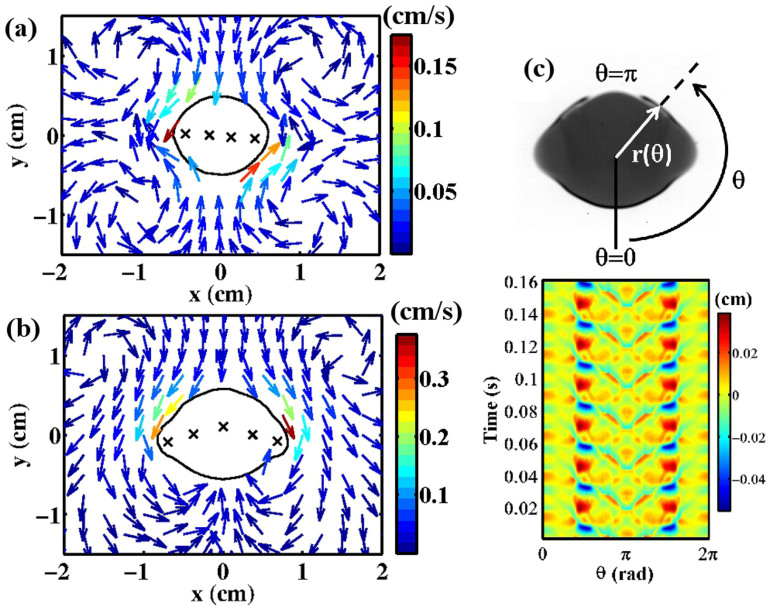
(a, b) Steady flow around droplet. The arrows indicate the flow direction and the color indicates the speed. Crosses indicate peak positions of the antinode. The coordinate axes are fixed on the droplet. (a) Spinning motion. The droplet spins clockwise. (b) Straight motion. The droplet migrates upward. (c) Color map image of the surface wave at the triple line. The color indicates the magnitude of the oscillation of the shape, *δr* = *r*(*θ*, *t*) − 〈*r*(*θ*, *t*)〉*_t_* (see Methods). For a straight motion, a standing surface wave is observed. (a–c) *ν* = 400 mm^2^/s, *V_d_* = 0.6 ml, *f* = 80 Hz. (a) Γ = 67 m/s^2^. (b, c) Γ = 72 m/s^2^.

We first measured the time-averaged velocity field of the silicone-oil bath around the droplet by particle tracking with tracers. For spinning, rotational, and straight motion, four vortices always appear near the silicone-oil-air interface (Figs. 3(a) and 3(b)). The silicone oil flows in along the short axis and flows out along the long axis of the droplet. Corresponding to the motion of the droplet, the symmetry of the vortices is broken. The vortices around the stationary elongated droplet have a symmetric shape. In contrast, for spinning motion, the two diagonal vortices rotate faster than the others. Thus, rotational symmetry is broken (Fig. 3(a)). For straight motion, the symmetry with respect to the long axis of the droplet is broken (Fig. 3(b)). The flow field indicates that the droplet is a “puller”^18^. In both cases, the droplet moves toward the faster vortices. A spatio-temporal plot of the oscillation of the droplet shape is shown in Fig. 3(c). Red and blue colors indicate the largest amplitude of the oscillation. Figure 3(c) shows that the amplitude of the oscillation is largest along the long axis, and a standing surface wave is generated at the triple line of the droplet by a Faraday wave. A similar result is obtained for spinning and rotational motion. This oscillation in shape gives rise to a corresponding oscillatory flow around the droplet. It was reported that oscillatory flow around a solid body or a bubble generates steady vortices^19^,^20^,^21^. If the vortices are asymmetrically shaped, they cause directional motion of a solid body^22^. Since the low-Reynolds-number approximation is weakly violated (*Re* is approximately 0.1), we numerically simulated the two-dimensional (2D) Navier-Stokes' equation by accounting for the oscillating boundary conditions observed in the experiment. We then reproduced the steady vortices around the droplet (see SI). Thus, for a standing surface wave, the oscillatory flow generates steady vortices. By considering conservation of momentum, the counteraction of the asymmetric vortices must be the driving force of the droplet^23^.

### Phase diagram and bifurcation of migratory motion

**Figure 4:**
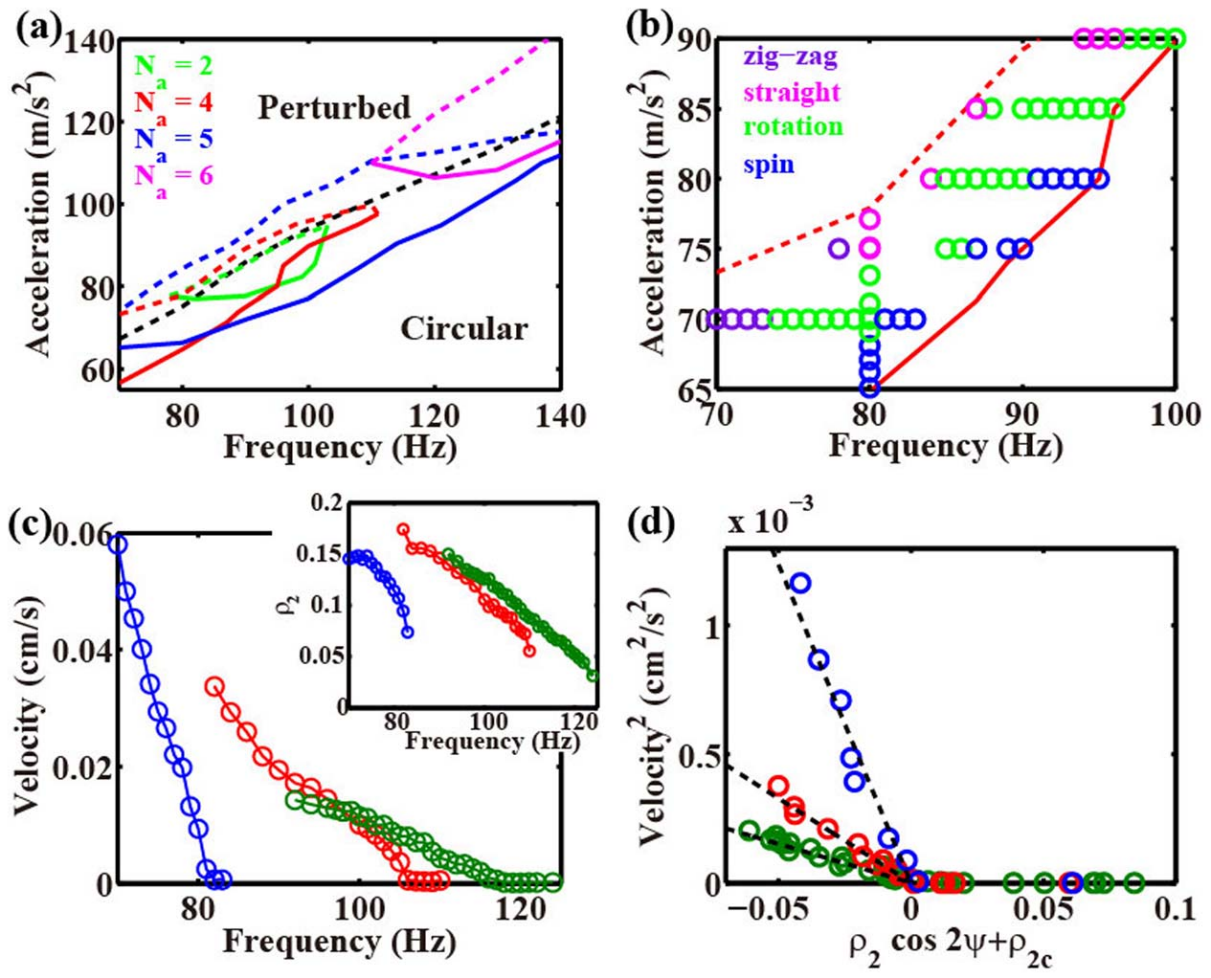
(a) Phase diagram of Faraday wave on droplet. Solid and dashed curves represent lower and higher limits for Faraday waves with a given *N_a_*, respectively (color indicates *N_a_*). Above (below) the dashed (solid) curve, a Faraday wave becomes unstable. Between the solid and dashed lines, a Faraday wave is stable. Green: *N_a_* = 2. Red: *N_a_* = 4. Blue: *N_a_* = 5. Magenta: *N_a_* = 6. Above the black dashed line, the circular shape (no Faraday resonance) is unstable. In the perturbed region, a steady Faraday wave is perturbed. (b) Phase diagram of motion of droplet for *N_a_* = 4. Red curves are identical to the boundaries of *N_a_* = 4 (Fig. 3(a)). Blue: spinning motion. Green: rotational motion. Magenta: straight motion. Purple: zigzag motion (see Fig. 4(a)). (c) Velocity of the droplet as a function of frequency. (Inset) *ρ*_2_ as a function of frequency. (d) Velocity squared as a function of *ρ*_2_cos 2*ψ*. As measured, the fitting parameter *ρ*_2c_ is always positive. We eliminated the data points for zigzag motion. (a–d) *ν* = 400 mm^2^/s, *V_d_* = 0.6 ml. (c, d) Blue: *N_a_* = 4, Γ = 70 m/s^2^. Red: *N_a_* = 5, Γ = 85 m/s^2^. Green: *N_a_* = 5, Γ = 95 m/s^2^.

Here, we show how the dynamics depends on *f* and *Γ*. First, we focus on the number *N_a_* of antinodes of Faraday waves and show a phase diagram based on *N_a_* (Fig. 4 (a)). Above the black dashed line, a Faraday wave appears on the circular droplet. Once the Faraday wave appears, it remains even if acceleration is reduced below the dashed line. Depending on the initial perturbation of the droplet, multiple *N_a_* can coexist in the same parameter region. For example, at *f* = 90 Hz and *Γ* = 80 m/s^2^ in Fig. 4(a), *N_a_* = 2, *N_a_* = 4, and *N_a_* = 5 are stable for small perturbation. Figure 4(b) shows a phase diagram of the motion for *N_a_* = 4. At high frequency and low acceleration, spinning motion is observed. As the frequency is reduced, drift bifurcation occurs and the spinning motion bifurcates to rotational motion. Zigzag and straight motions are observed at low frequency and high acceleration. As *N_a_* increases, the phase diagram shifts to a higher frequency and higher acceleration. As the viscosity *ν* decreases, irregular polygonal turning and squirming motion become predominant.

**Figure 5:**
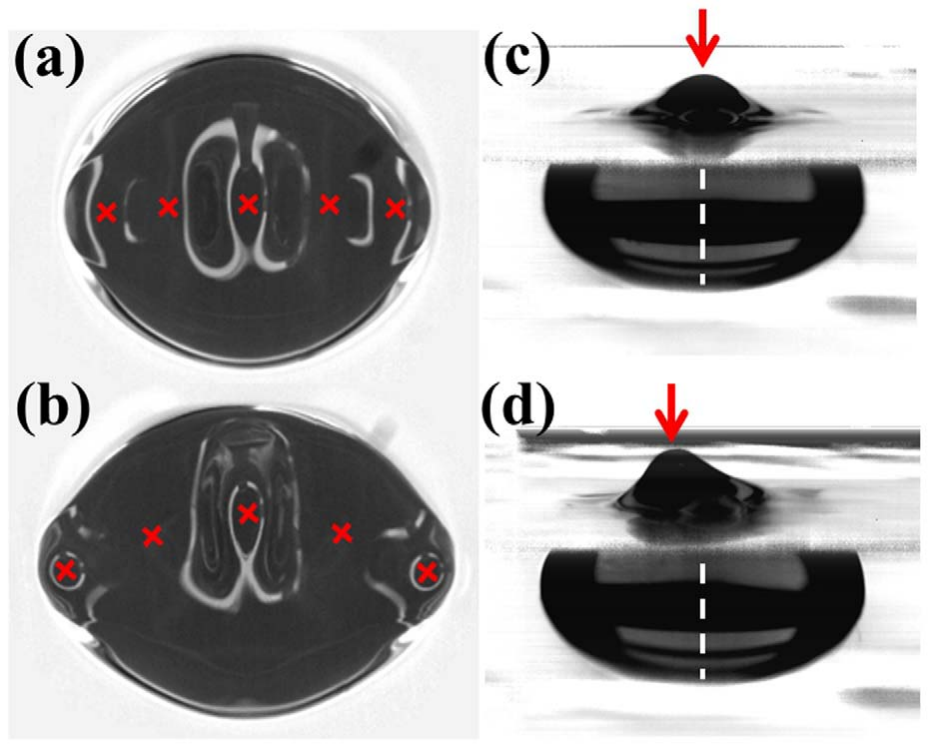
(a, b) Top view of droplets around drift bifurcation. Red crosses indicate the position of the antinode. (c, d) Lateral view of a droplet around drift bifurcation. The horizontal axis is parallel to the short axis of the droplet. Red arrow indicates the peak position of the antinode. White dashed line indicates the center of the droplet. (a) Droplet before drift bifurcation. Positions of the antinodes align (red crosses). The droplet spins very slowly and is almost stationary. *f* = 111 Hz. (b) Droplet after drift bifurcation. The antinodes near the centroid of the droplet move forward (red crosses). The droplet migrates upward. *f* = 91 Hz. (c) Droplet before drift bifurcation. *f* = 107 Hz. (d) Droplet after drift bifurcation. The antinode moves forward (red arrow). The droplet migrates leftward. *f* = 91 Hz. (a–d) *ν* = 400 mm^2^/s, *V_d_* = 0.6 ml, *N_a_* = 5, Γ = 85 m/s^2^.

Next, we analyze the bifurcation from spinning motion to rotational motion with a particular focus on the slow dynamics. We calculate the time-average of the velocity of the centroid *V* and the magnitude of the elliptical deformation *ρ*_2_ as a function of frequency (see Methods). The wave length *λ* of the Faraday wave is a decreasing function of frequency^17^,^24^. For an elongated droplet, *ρ*_2_ is approximated by *N_a_λ*/(4*R*) −1 on the basis of a geometric requirement. Therefore, as the frequency is reduced, *λ* and *ρ*_2_ increase, and the droplet is strongly deformed (Fig. 4(c), inset). As shown in Fig. 4(c), a critical frequency exists below which drift instability appears. When drift bifurcation occurs, the symmetry of the Faraday wave is simultaneously broken. For the stationary and spinning droplet, the peak positions of the antinode are aligned in a straight line (Fig. 5(a), red crosses and Fig. 5(c), red arrow). Once drift bifurcation occurs, the antinodes near the centroid of the droplet move forward (Fig. 5(d), red arrow) and a curve that connects the peak positions of the antinode gets a forward curvature (Fig. 5(b), red crosses).

We now summarize the process of drift bifurcation. Above the critical frequency, the wave length and elongation of the droplet merely increases as the vibration frequency deceases. Below the critical frequency, the peak positions of the antinode starts to move forward and the droplet begins to migrate (Fig. 5(d), red arrow). A similar bifurcation process occurs when we increase the acceleration with fixed vibration frequency. For as long as we observed, drift bifurcation and the curved antinode position simultaneously occur as the elongation of the droplet increases. Even if we restrict droplet migration by fixing it to the bottom of the container, aligned antinodes become unstable and antinode positions start to have a forward curvature as the elongation becomes large. Thus, regardless of whether the droplet migrates, symmetry breaking of the Faraday wave occurs. The radiation pressure of the Faraday wave causes the deformation of the droplet^16^,^17^. As mentioned before, oscillatory flow and consequent steady streaming are due to the Faraday wave. Therefore, spontaneous symmetry breaking of the Faraday wave should induce an asymmetric shape of the droplet and steady vortices, followed by migration. This suggests that symmetry breaking of the shape causes migration, and not the other way around.

### Analytical model of droplet

We propose a phenomenological model of a droplet based on a model of a self-propelled particle with coupling of the velocity and shape tensor^25^. Because the migration of the droplet is restricted to 2D, we use equations based on a 2D model. The time-scale of the motion of the droplet is approximately10s, which is thousands of times longer than a cycle of a Faraday wave (approximately 0.01s). For a deformable migrating particle, the dynamics equations for slow variables *V* and *ρ*_n_ (n = 2, 3, 4,…) can be derived by considering the spatial symmetry^25^,^26^,^27^. A generic form of the equations is shown in the SI. Here, we only consider small deformation and neglect higher modes *ρ*_n_ (n > 2). To describe drift bifurcation, the minimum set of evolution equations of *V* and elliptic deformation *ρ*_2_ is 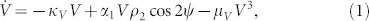

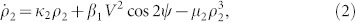
where *κ*_v_, *κ*_2_, *α*_1_, *β*_1_, *μ*_v_, and *μ*_2_ are constant coefficients, and *ψ* = *ϕ*_2_ − *ϕ_v_* (see Methods). Since we consider steady elongated deformation, *κ*_2_ > 0. In addition, we assume that *μ*_v_ and *μ_2_* are also positive because no hysteresis occurs around the drift bifurcation. For small elongation *ρ*_2_, *V* = 0 is a stable solution. As the elongation *ρ*_2_ increases, the solution *V* = 0 becomes unstable and a supercritical pitchfork bifurcation occurs. *V* has the stable solution 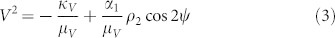
The experimental data can be fit by Eq. (3) with fitting parameters *γ* = *α*_1_/*μ*_v_ and *ρ*_2*c*_ = −*κ*_v_/*α*_1_, where *γ* is the slope and *ρ*_2c_ is the intercept (Fig. 4(d)). Since we vary the frequency *f* along each curve, this expression must be independent of *f*. By fitting the curves in Fig. 4(d), we obtain *γ* < 0 and *ρ*_2c_ > 0. Consequently, *α*_1_ <0 and *κ*_v_ > 0 have to be satisfied in the model. *κ*_v_ > 0 indicates that the droplet does not undergo self-propulsion. Instead, because of the coupling term, migration is caused by strong elongation, as expected from the above discussion^28^.

### Complex motion of droplet both in the experiment and simulation

In addition to simple motions such as spinning, rotating and straight motions, more complex dynamics are also observed, such as zigzag motion, reciprocal motion, or irregular polygonal turning.

### Zigzag motion

**Figure 6:**
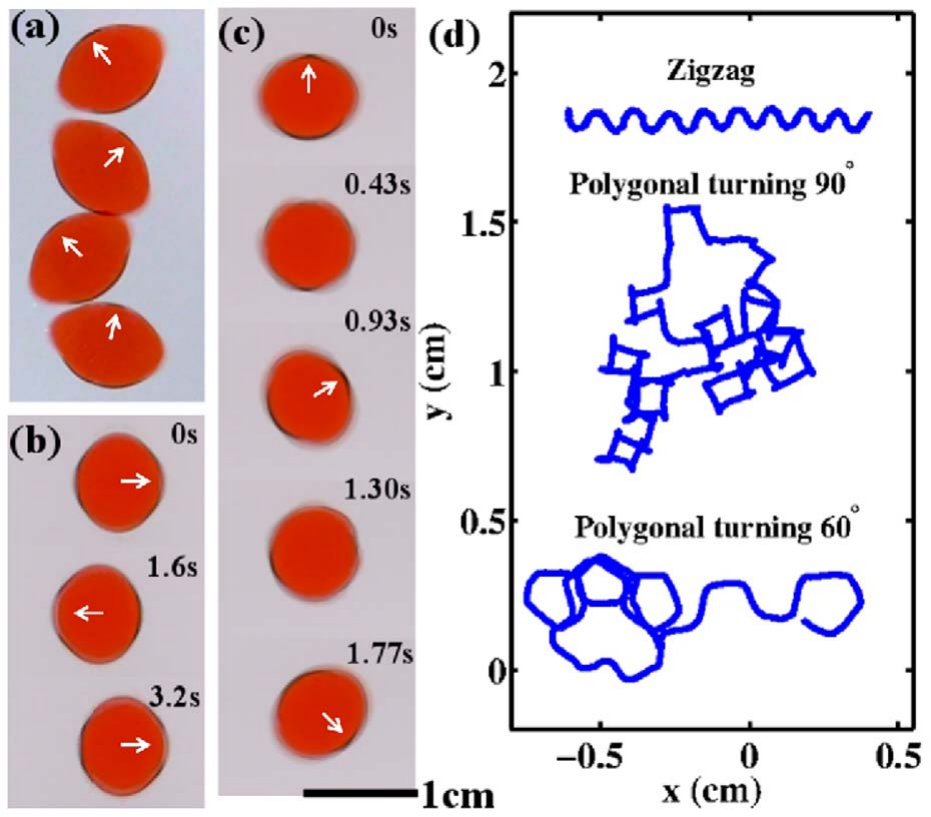
Top view of complex motions. Time-lapse images of (a) zigzag motion. Time series of (b) reciprocating motion, and (c) irregular polygonal turning. (d) Trajectory of centroid for zigzag motion and irregular polygonal turning. For irregular polygonal turning, two trajectories (turning angles of 60° and 90°) are shown. (a) *ν* = 400 mm^2^/s, *f* = 93 Hz, *V_d_* = 0.3 ml, Γ = 111 m/s^2^. (b) *ν* = 37 mm^2^/s, *f* = 90 Hz, *V_d_* = 0.3 ml, Γ = 74 m/s^2^. (c) *ν* = 37 mm^2^/s, *f* = 120 Hz, *V_d_* = 0.3 ml, Γ = 95 m/s^2^.

The droplet undergoes translational motion, but both the direction of the long axis and the velocity oscillate simultaneously (Fig. 6(a)). Depending on the frequency and acceleration, the amplitude of the oscillation varies continuously (typically, 7° to 70°). Consequently, the trajectory of the centroid follows a zigzag curve (Fig. 6(d)). Zigzag motion can be found with a high acceleration, a low frequency, and a highly viscous silicone-oil bath (Fig. 4(b)).

### Reciprocal motion

The droplet moves periodically back and forth. Asymmetric deformation with respect to the long axis also oscillates (Fig. 6(b)).

### Irregular polygonal turning

The short and long axes periodically change their directions by certain angles (60° or 90°; see Fig. 6 (c)). The droplet temporarily has a circular shape when the axes change directions. The droplet migrates in the direction of the short axis. Thus, the trajectory of the centroid is a polygonal line (Fig. 6(d)). Irregular polygonal turning is observed mainly in low-viscosity silicone oils.

**Figure 7:**
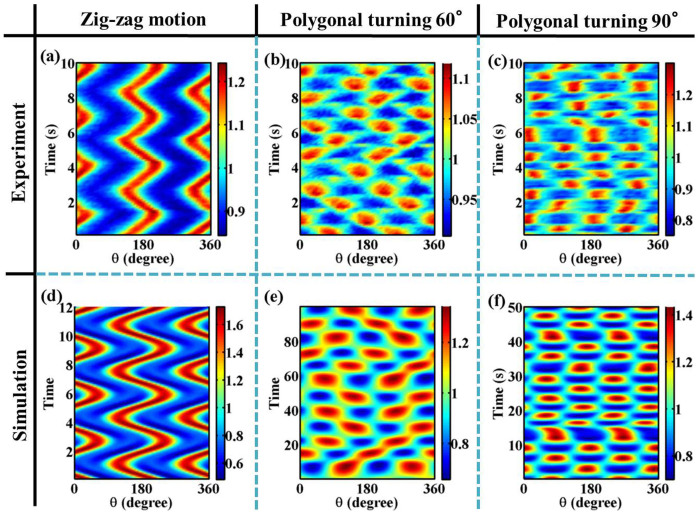
Spatio-temporal plots of *r*(*θ*, *t*) normalized by the radius. In this figure, we show the slow dynamics of the shape. Red and blue colors represent the long and short axes, respectively. (a–c) Experimental results. (d–f) Simulation results. (a, d) Zigzag motion. (b, e) Irregular polygonal turning. Turning angle is 60°. (c, f) Irregular polygonal turning with a turning angle of 90°. (a) *ν* = 400 mm/s^2^, *f* = 93 Hz, *V_d_* = 0.3 ml, Γ = 111 m/s^2^. (b) *ν* = 37 mm/s^2^, *f* = 120 Hz, *V_d_* = 0.3 ml, Γ = 95 m/s^2^. (c) *ν* = 37 mm/s^2^, *f* = 90 Hz, *V_d_* = 0.6 ml, Γ = 82 m/s^2^, and the temperature is 17.2°C. (d–f) The parameter values are defined in the SI. (d) The parameter values are *κ*_v _ = 0.3, *α*_1_ = −2.0, *α*_3_ = 8.0, *μ*_v_ = 1.0, *κ*_2_ = 1.5, *β*_1_ = 1.0, *β*_3_ = −8.0, *β*_4_ = −8.0, *μ*_2_ = 1.0, *κ*_4_ = 2.0, *λ*_1_ = 1.75, *λ*_2_ = −0.0625, the other values are zero. (e) *κ*_v_ = 0.14, *α*_1_ = −1.0, *α*_2_ = 0.188, *μ*_v_ = 4.0, *κ*_2_ = 0.12, *β*_1_ = 0.04, *β*_2_ = −6.0, *μ*_2_ = 1.0, *κ*_3_ = 0.05, *ν*_1_ = 0.3, *μ*_3_ = 4.0, the other values are zero. (f) *κ*_v_ = 0.11, *α*_1_ = −2.0, *α*_3_ = 4.0, *μ*_v_ = 1.0, *κ*_2_ = 0.4, *β*_1_ = 1.0, *β*_3_ = −4.0, *β*_4_ = −4.0, *μ*_2_ = 1.0, *κ*_4_ = 0.3, *λ*_1_ = 0.25, *λ*_2_ = −0.125, the other values are zero.

We find that all motions observed in the experiments can be reproduced by equations based on the phenomenological model of a deformable particle by considering a fourth-order tensor^27^. The equations are derived only from the symmetry argument and have the general form up to the third order nonlinear term^25^,^26^,^27^,^28^. The model equations and phase diagram obtained from the numerical calculation are shown in the Supplementary Information. Spatio-temporal plots of zigzag motion and irregular polygonal turning are shown in Figs. 7(a)–(f). The color in Figs. 7(a)–(f) indicates the shape *r*(*θ*, *t*) of the droplet, and red and blue colors represent the long and short axes, respectively. For zigzag motion (Fig. 6(a)), the numerical simulation successfully reproduces oscillations in the direction of the long axis (Figs. 7(a) and (d)) and the zigzag trajectory of the centroid (see SI). The numerical simulation also produces irregular polygonal turning, where the short and long axes periodically change directions by certain angles (Figs. 7(b), (c), (e), and (f)). The trajectory of the centroid follows a polygonal line (see SI). In the experiment, irregular polygonal turning appears at low viscosity. In the simulation, irregular polygonal turning appears at a small *κ*_v_ value of approximately 0.05. Since the term with *κ*_v_ reflects the viscous drag force, this simulation result is consistent with the experimental observation. For a larger *κ*_v_ value of approximately 0.5, the dynamics changes from spinning motion to rotational motion to zigzag motion as *κ*_2_ increases. In the experiment, as the elongation of the droplet increases, the same sequence of bifurcations occurs at high viscosity. Since the magnitude of the elongation is an increasing function of *κ*_2_, the proposed model's equations qualitatively reproduce the cascade of mode bifurcations.

## Discussion

In this work, we observe a swimming droplet driven by Faraday waves and find a cascade in the bifurcations of the motion. The experimental observations indicate that symmetry breaking of the Faraday wave is crucial for the droplet migration. Thus, an important problem that remains is to elucidate the mechanism of the spontaneous symmetry breaking of the Faraday wave with an elongated flexible boundary. In our phenomenological model, we assume that only the elongation mode is “active” and other modes, including the drift mode, are “passive”. Thus, no distinguishing motion arises at small elongation. It is non-trivial that strong elongation leads to a cascade in the bifurcations of the motion, as experimentally observed. However, because the model is derived only from the spatial symmetry, relationships between coefficients in the model and physical parameters are uncertain. To reveal the physical meaning of the coefficients, a calculation based on hydrodynamics is essential^29^. Resolution of these questions should lead to deeper understanding of the important process of migration of soft active particles.

The system of a droplet with a Faraday wave is very simple, but the droplet shows rich dynamics that resembles those of living organisms^30^ and those predicted by theoretical works^28^. This should enable us to understand the spontaneous motion of living organisms. The collective behavior of living organisms^31^ and active colloids^32^,^33^,^34^ has recently been extensively investigated in physics. The dynamics of these active objects is considered a fundamental subject of statistical physics far from equilibrium. The motion of swimming droplets is easy to control and hence our system can be used; however, extending our system to collective behavior would be challenging. Water droplets with surface waves are promising candidates for a prototypical system that can be used to investigate the full landscape of the dynamics of soft active particles.

## Methods

### Experimental setup

A layer of silicone oil (1-cm deep) in an acrylic container (9-cm-inner diameter) was subjected to vertical sinusoidal vibration [vertical position *z*(*t*) = *A*sin (2*π* *f t*)] using an electromagnetic vibration system. A flat LED panel was placed between the acrylic container and the vibrator, and the droplet was illuminated from below. The control parameters of the system were vibration frequency *f*, vibration acceleration *Γ* = *A* (2*π* *f*)^2^, viscosity *ν* of the silicone-oil bath, and volume *V_d_* of the droplet. The surface tension between the dyed water and air was 69.6 mN/m, and the surface tension between the dyed water and silicone oil was approximately 50 mN/m. The surface tension between silicone oil and air was 21 mN/m. The frequency was varied from 60 to 140 Hz, and the peak acceleration was varied up to 150 m/s^2^. The densities and kinematic viscosities of silicone oil are 1.065 g/cm^3^ (*ν* = 37 mm/s^2^), 1.06 g/cm^3^ (*ν* = 170 mm/s^2^), and 1.07 g/cm^3^ (*ν* = 400 mm/s^2^). Unless otherwise stated, the temperature was between 26.0°C – 27.0°C.

### Analytical method

Because the motion of the droplets is restricted to 2D, we analyzed the top view of the droplets. From a binarized top-view image, the centroid *x*(*t*) of the droplet was measured. The distance from the centroid to the edge of the droplet was then measured as *r*(*θ, t*), where the angle *θ* is measured with respect to the *x* axis. The quantity *r*(*θ, t*) can be represented as a Fourier series: 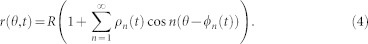
The normalized amplitude *ρ_n_* determines the shape of the droplet. Elliptical deformation is represented by the mode *n* = 2, and *ϕ*_2_ is the angle between the long axis of the ellipsoid and the *x* axis. We also measured the speed of the centroid *V* and the direction of motion *ϕ*_v_ measured from the *x* axis.

Here, we define the magnitude of the oscillation of the droplet shape, 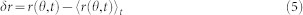


Using *δr*, we calculate the *Re* as 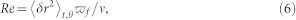
where *ω_f_* is the angular frequency of the oscillation.

## Author Contributions

H.E. conducted all experiments, derivations, computations, and analyses. M.S. proposed the project, directed the research, and proposed the analyses. H.E. and M.S. discussed the results and wrote the paper.

## Supplementary Material

Supplementary Information Supplementary Information

Supplementary Information Top view of the simple motions of a droplet

Supplementary Information Top view of the complex motions of a droplet

Supplementary Information Lateral view of a droplet with spinning motion

Supplementary Information Top-view image of squirming motion

Supplementary Information Top-view image of straight motion
