# Supplementary material for: Swimming droplets driven by a surface wave
Source: Sci Rep. 2015 Feb 24;5:8546. doi: 10.1038/srep08546 (PMC4338424; doi:10.1038/srep08546)
Supplement: Supplementary Information [file srep08546-s1.pdf]

Supplementary Information for
"Swimming droplets driven by a surface wave"

Hiroyuki Ebata[1] & Masaki Sano[2]

[1]Department of Physics, Graduate School of Science, Chiba University, Yayoi-cho, Chiba, 263-8522, JAPAN
[2]Department of Physics, Graduate School of Science, The University of Tokyo, Hongo, Tokyo 113-0033, JAPAN

A. Movie legends

simple_motions.mov

Top view of the simple motions of a droplet induced by a Faraday wave. The video contains straight motion, rotational motion, and spinning motion. Parameter values are shown in the video. Screen width corresponds to 5.6cm.

complex_motions.mov

Top view of the complex motions of a droplet induced by a Faraday wave. The video contains squirming motion, zigzag motion, reciprocal motion, and irregular polygonal turning at 60 degrees. Parameter values are shown in the video. Screen width corresponds to 5.6cm.

stationary_hispeed.mov

Lateral view of a droplet with spinning motion captured by high-speed camera. The horizontal axis is parallel to the long axis of the droplet. The video was acquired at 1200 frames / sec and was edited to play 60x slower than real time. Screen width corresponds to 1.6cm. Parameter values are identical to that of Fig. 2 (d) in the main text.

squirm_hispeed.mov

Top-view image of squirming motion captured by a high-speed camera. The video was acquired at 1000 frames / sec and was edited to play 120x slower than real time. Screen width corresponds to 2.1cm. Parameter values are identical to that of Fig. 3 (c) in the main text.

straight_hispeed.mov

Top-view image of straight motion captured by a high-speed camera. The video was acquired at 1200 frames / sec and was edited to play 60x slower than real time. Screen width corresponds to 1.7cm. Parameter values are identical to that of Fig. 2 (g) in the main text.

B. Traveling wave of squirming motion

The traveling wave is generated at the water-oil-air triple line. Thus, a squirming droplet swims due to the traveling wave. A spatio-temporal plot of the droplet with the traveling wave is shown in Fig. S1 (a). The color indicates the magnitude of deformation. Red and blue represent the peak and bottom of the traveling wave, respectively. As shown in Fig. S1 (a), the traveling wave periodically propagates from the "head" to the "tail" of the droplet at nearly the same frequency as the Faraday wave (typically 50Hz). In addition, the Faraday wave slowly propagates from the "tail" to the "head" along the long axis of the droplet (typically 3Hz), and nodes and anti-nodes are sequentially generated at the "tail" and disappear at the "head" (Fig. S1 (b)).

C. Model of the deformable particle

The time-evolution equation for deformable particles is derived in [S1 - S3]. Equivalent equations can also be derived from symmetry arguments [S4]. By using a Fourier series, the deformation of a 2D particle around a circular shape can be written as

$$r(\theta,t) = 1 + \sum_{n=-\infty}^{\infty} z_n(t)e^{in\theta} ,$$

where we set the radius to be unity. Complex amplitude z_n can be expressed as $z_n=\rho_n\exp(in\phi_n)$. ρ_n and ϕ_n are identical to those in the Methods. Here, we define the following complex velocity:

$$\mathrm{v}_1 = V\exp(i\phi_v) ,$$

where V and ϕ_v are the speed of the centroid and the direction of migration, respectively. For convenience, we set z_1 = v_1 and z_{-n} as the complex conjugate of z_n. The general time-evolution equations for z_n (n is a positive integer) are derived by considering possible coupling terms up to the third order [S4].

$$\frac{dz_n}{dt} = A_n z_n + \sum_{i+j=n} B_{ij} z_i z_j + \sum_{i+j+k=n} C_{ijk} z_i z_j z_k ,$$

where i, j, and k are integers excluding 0, and A_i, B_{ij}, and C_{ijk} are real constant coefficients.

Here, we found that all of the observed motions in the experiment can be described by model equations of deformable particles by considering a 4th order rank tensor [S3]. For simplicity, we only consider the terms that seem to be important for mode bifurcation.

$$\frac{d\mathrm{v}_1}{dt} = -\kappa_{\mathrm{v}}\mathrm{v}_1 + \alpha_1 z_2 \mathrm{v}_{-1} + \alpha_2 z_3 z_{-2} + \alpha_3 \mathrm{v}_{-1} z_{-2} z_4 - \mu_v \mathrm{v}_1 |\mathrm{v}_1|^2 , \quad \text{(S1)}$$

$$\frac{dz_2}{dt} = \kappa_2 z_2 + \beta_1 \mathrm{v}_1^2 + \beta_2 z_3 \mathrm{v}_{-1} + \beta_3 z_4 z_{-2} + \beta_4 \mathrm{v}_{-1}^2 z_4 - \mu_2 z_2 |z_2|^2 , \quad \text{(S2)}$$

$$\frac{dz_3}{dt} = -\kappa_3 z_3 + \nu_1 \mathrm{v}_1 z_2 - \mu_3 z_3 |z_3|^2 , \quad \text{(S3)}$$

$$\frac{dz_4}{dt} = -\kappa_4 z_4 + \lambda_1 z_2^2 + \lambda_2 \mathrm{v}_1^2 z_2 , \quad \text{(S4)}$$

where v_{-1} is a complex conjugate of v_1, and κ_i, α_i, β_i, ν_i, λ_i, and μ_i (i = v, 1, 2, 3, 4) are real constant coefficients. Here we set $\kappa_i > 0$ (i = v, 1, 2, 3, 4). Thus, we assume that a circular shape is linearly unstable, and elongation always occurs. In the experiment, $\rho_2 > \rho_4 >> \rho_3$ is satisfied in many cases.

First, we show the numerical results of the equations for v_1, z_2 and z_4, while ignoring z_3. Thus, we only consider Eqs. (S1), (S2) and (S4) with $\alpha_2 = \beta_2 = 0$. The phase diagram obtained from the numerical calculation is shown in Fig. S2 (a). Spinning motion (blue open circle), straight motion (magenta open circle), rotational motion (green open circle), zigzag motion (purple open circle) and polygonal turning at 90 degrees (red open square) are seen. For small κ_v and large κ_2, the dynamics become highly complex including irregular polygonal turning and chaotic motions, and we do not show these dynamics in Fig. S2 (a). The trajectories of zigzag motion and irregular polygonal turning at 90 degrees are shown in Figs. S3 (a), (c). In addition to the above basic motions, we found quasi-periodic motions. The filled magenta circles show straight motion, but V and ρ_2 oscillate. Filled green circles and filled red squares show quasi-periodic rotational motion [S2] and quasi-periodic polygonal turning. These motions have two frequencies with an irrational ratio. Therefore, the trajectories of the centroid do not close.

Around the bifurcation from spinning motion to rotational motion, V^2 becomes an almost linear function of $\rho_2 \cos 2\psi$ (Fig. S2 (b)). As long as ρ_2 is not so large, z_3 and z_4 are negligible. If we omit the terms z_3 and z_4, Eq. (S1) and (S2) can be transformed into Eqs. (1) and (2) in the main text. Thus, Fig. S2 (b) approximately satisfies Eq. (3) in the main text.

Next, we show the numerical results of the equations for v_1, z_2 and z_3, while ignoring z_4. Thus, we only consider Eqs. (S1), (S2) and (S3) with $\alpha_3 = \beta_3 = \beta_4 = 0$. We found reciprocal motion and polygonal turning at 60 degrees. The trajectory of irregular polygonal turning at 60 degrees is shown in Fig. S3 (b). Figure S3 (d) shows the time series of reciprocal motion.

D. Simulation of the 2D Navier-Stokes equation

To determine the relation between oscillatory flow and steady flow in the silicone oil, we simulate the 2D Navier-Stokes equation. Before the simulation, we measure the oscillatory flow near the droplet-silicone oil interface of the stationary elongated droplet. The flow near the droplet is measured by particle-tracking using tracers. The particles are lighter than the silicone oil and float near the air – oil interface. Thus, we measure only the horizontal component of the flow near the air – oil interface. As shown in Figs. S4 (a) and (b), flow speed is greatest near the long axis of the droplet, and the flow oscillates between incoming flow and outgoing flow. This flow field indicates that the volume of a droplet immersed in the silicone oil is not preserved and instead oscillates. Next, we focus on the flow along the droplet – oil interface. We measure the velocity 1.2mm from the droplet interface. The velocity is decomposed into a tangential component $V_t(t,\theta)$ and a normal component $V_n(t,\theta)$ with respect to the droplet-oil interface (see schematic illustration, Fig.S4 (c)). Here, θ is defined around the centroid and is the angle with respect to the long axis. We found that $V_n(t,\theta)$ is several times larger than $V_t(t,\theta)$, and the spatio-temporal plot of $V_n(t,\theta)$ is shown in Fig. S4 (d). Figure S4 (d) indicates that strong oscillatory flow is localized around the long axis, and the flow almost becomes a standing wave.

By using the experimental data on oscillatory flow, we simulate the two-dimensional flow of the Navier-Stokes equation. As a boundary condition on the droplet, we use the oscillatory flow $V_t(t,\theta)$ and $V_n(t,\theta)$ which are obtained in the experiment. Next, we check whether the steady flow near the droplet is reconstructed or not. In the simulation, we ignore deformation of the droplet and flow in the droplet. The parameter values are chosen to be the same as those in the experiment.

We calculate the 2D Navier Stokes equation with polar coordinates:

$$\partial_t v_r + (\vec{v}\cdot\nabla)v_r - \frac{v_\theta^2}{r} = -\frac{1}{\rho}\partial_r p + \nu\left(\Delta v_r - \frac{2}{r^2}\partial_\theta v_\theta - \frac{v_r}{r^2}\right),$$

$$\partial_t v_\theta + (\vec{v}\cdot\nabla)v_\theta + \frac{v_r v_\theta}{r} = -\frac{1}{\rho r}\partial_\theta p + \nu\left(\Delta v_\theta + \frac{2}{r^2}\partial_\theta v_r - \frac{v_\theta}{r^2}\right).$$

The equation of continuity is

$$\frac{1}{r}\partial_r(r v_r) + \frac{1}{r}\partial_\theta v_\theta = 0.$$

At the surface of the droplet $r=R$,

$$v_r = V_n(t,\theta),\quad v_\theta = V_t(t,\theta),\quad p = -\frac{\sigma}{R} + 2\nu\partial_r v_r,$$

where R and σ are the radius of the droplet and the surface tension, respectively. At the boundary far from the droplet $r=r_F$,

$$\partial_r v_r = 0, \quad \partial_r v_\theta = 0, \quad p = -\frac{\sigma}{R},$$

where we set a constant pressure to eliminate the mean flow.

In the simulation, we obtained the four vortices of steady streaming as seen in the experiment (Fig.S4 (e)). Compared to the experiment, the speed of the vortices is slightly small near the droplet and large at a distance from the droplet. This difference could be due to the two-dimensionality of the simulation. We introduce fitting parameter α and substitute $\alpha V_t(t,\theta)$ and $\alpha V_n(t,\theta)$ at the boundary condition. Then, we found that we could fit the experimental results well (Fig. S4 (f)). These results indicate that steady streaming is generated by oscillation of the triple line.

[S1] T. Ohta and T. Ohkuma, Phys. Rev. Lett. **102** 154101 (2009)

[S2] T. Hiraiwa, M. Y. Matsuo, T. Ohta and M. Sano, EPL **91** 20001 (2010)

[S3] M. Tarama and T. Ohta, Phys. Rev. E, **87** 062912 (2013)

[S4] D. Armbruster, J. Guckenheimer and P. Holmes, SIAM J. Appl. Math. **49** 676 (1989)

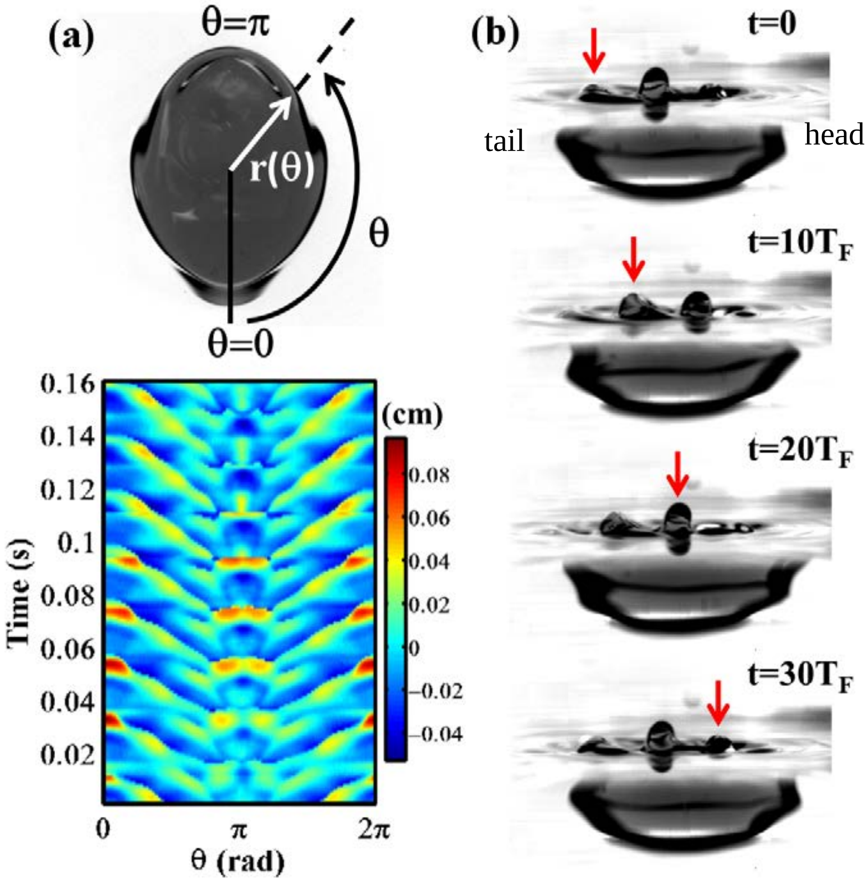

Fig. S1: (a) Color map image of the surface wave at the triple line. The color indicates the magnitude of deformation, $\delta r = r(\theta,t) - \langle r(\theta,t)\rangle_t$. $\theta = \pi$ indicates the "head" and $\theta = 0$ indicates the "tail". In the case of squirming motion, a traveling surface wave is observed. (b) Lateral view of the droplet with squirming motion. The horizontal axis is parallel to the long axis of the droplet. The droplet migrates from left to right. The arrow indicates the time series of one anti-node, which propagates slowly. $T_F = 2/f = 0.02$s. (a, b) $\nu = 37$mm^2/s, $f = 100$Hz, V_d =0.66ml. (a) Γ =79m/s^2, (b) Γ = 85m/s^2.

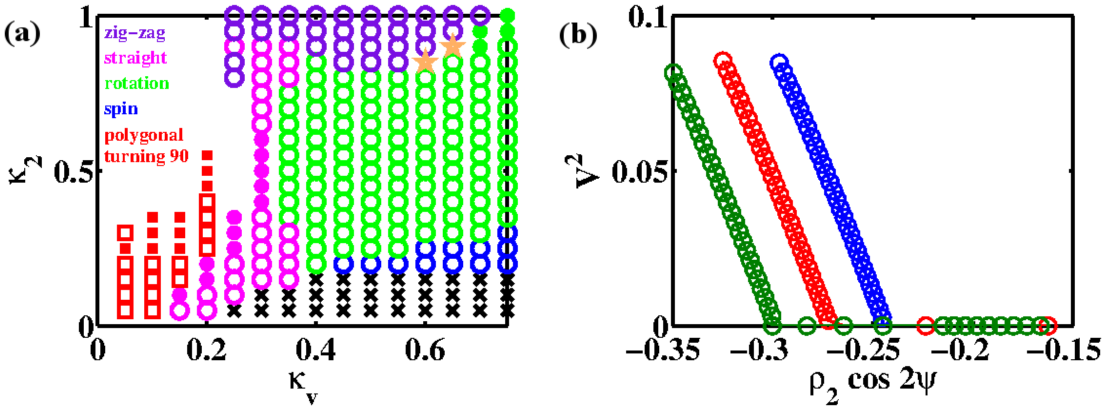

Fig. S2: (a) Phase diagram obtained numerically from Eq. (1) while ignoring the z_3 term. Cross: stationary elongation. Blue circle: spinning motion. Green circle: rotational motion. Green filled circle: quasi-periodic rotation. Magenta circle: straight motion. Magenta filled circle: straight motion with an oscillatory amplitude. Purple circle: zigzag motion. Red square: polygonal turning at 90 degrees. Red filled square: quasi-periodic polygonal turning at 90 degrees. Star: polygonal turning at 45 degrees. (b) Relation between V^2 and $\rho_2 \cos 2\psi$ around the bifurcation from spinning motion to rotational motion. ψ is defined as $\psi = \phi_2 - \phi_v$. Blue: κ_v=0.45. Red: κ_v=0.50. Green: κ_v=0.55. (a,b) The parameter values are α_1=-2.0, α_3=4.0, μ_v=1.0, β_1 =1.0, β_3=-4.0, β_4=-4.0, μ_2=1.0, κ_4=0.3, λ_1=0.25, λ_2=-0.125. Other values are set to be 0.

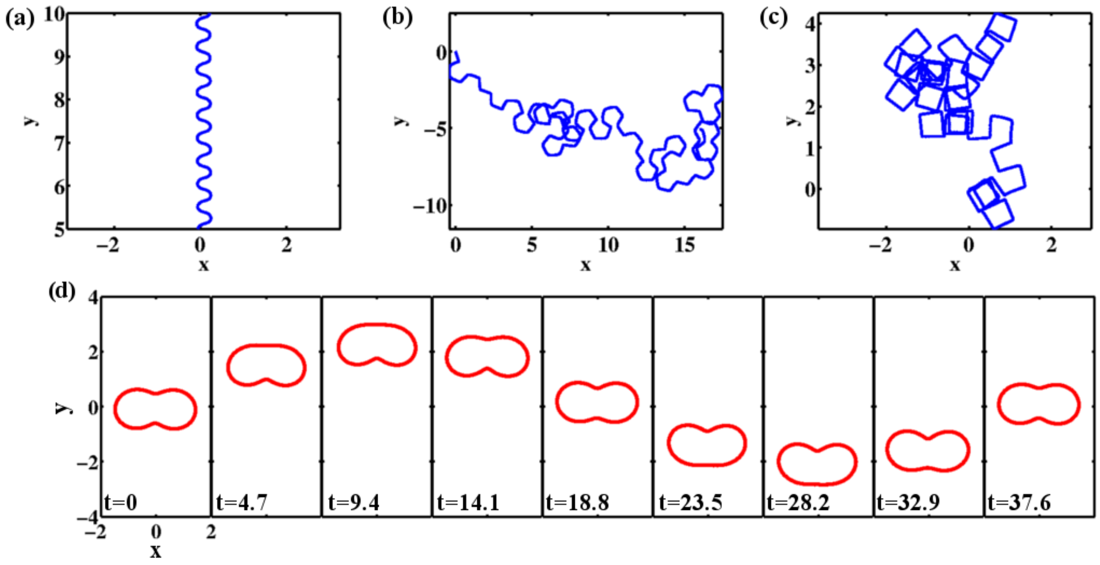

Fig. S3: (a,b,c) Trajectory of the centroid. (a) Zigzag motion. (b) Irregular polygonal turning at 60 degrees. (c) Irregular polygonal turning at 90 degrees. (d) Time series of reciprocal motion. (a, b, c) The parameter values are the same as those in Fig. 7 (e, f, g) in the main text. (a) κ_v=0.3, α_1=-2.0, α_3=8.0, μ_v=1.0, κ_2=1.5, β_1 =1.0, β_3=-8.0, β_4=-8.0, μ_2=1.0, κ_4=2.0, λ_1=1.75, λ_2=-0.0625. Other values are set to be 0. (b) κ_v=0.14, α_1=-1.0, α_2=0.188, μ_v=4.0, κ_2=0.12, β_1 =0.04, β_2=-6.0, μ_2=1.0, κ_3=0.05, ν_1=0.3, μ_3=4.0. Other values are set to be 0. (c) κ_v=0.11, α_1=-2.0, α_3=4.0, μ_v=1.0, κ_2=0.4, β_1 =1.0, β_3=-4.0, β_4=-4.0, μ_2=1.0, κ_4=0.3, λ_1=0.25, λ_2=-0.125. Other values are set to be 0. (d) κ_v=0.3, α_1=-1.0, α_2=1.5, μ_v=1.0, κ_2=0.2, β_1 =0, β_2=-1.5, μ_2=1.0, κ_3=0.02, ν_1=0.1, μ_3=1.0. Other values are set to be 0.

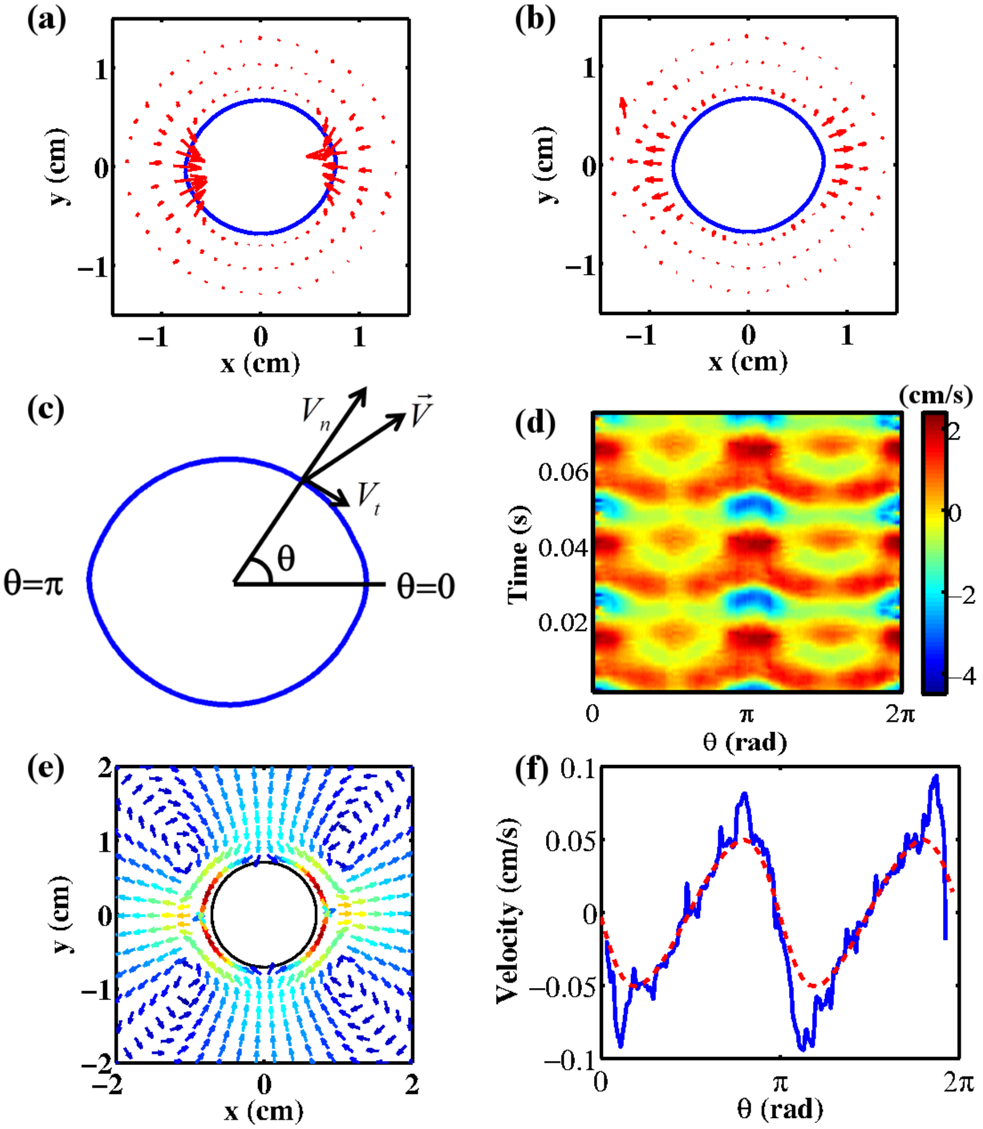

Fig. S4: (a,b) Oscillatory flow (red arrow) near the droplet (blue line). Flow oscillates between incoming flow ((a), t=0) and outgoing flow ((b), t=T/2), where T is the cycle of the Faraday wave. (c) Schematic illustration of the analysis. Blue curve shows the time-averaged shape of the droplet. (d) Time-space plot of $V_n(t,\theta)$. Red color indicates outgoing flow and blue color indicates incoming flow to the droplet. (e) Time-averaged velocity field obtained from the simulation. (f) Time-averaged tangential velocity $\langle V_t(t,\theta)\rangle_t$. The blue curve shows experimental data, and the red dashed curve is calculated from the simulation. In the simulation, the input velocity for the droplet is 1.71 times larger than the actual value. (a-d,f) ν = 400mm/s^2, f=80Hz, V_d=1.2ml, Γ=50m/s^2. (e,f) R=0.71cm, r_F =5.71cm, ν = 400mm/s^2.
